# Medical students at LTC and in pre-hospital care: a structured programme in pre-hospital emergency medicine for medical undergraduates

**DOI:** 10.1186/1757-7241-20-S1-I5

**Published:** 2012-03-22

**Authors:** George FA Kay, Enhui Yong, Sara A Dahlén, Anne Weaver

## Introduction

Pre-hospital emergency medicine is an emerging subspecialty in which medical undergraduates traditionally often receive little or no formal training. This article describes one academic programme at a London medical school, which comprises of a partnership between the school, the local ambulance and the air ambulance service. It is believed that this programme is unique in its structure and execution. Previously published material highlights only shorter courses or elective-based programmes. Students from the Pre-hospital Care Programme (PCP) assisted and participated in a pre-hospital student event which took place on the first student day of the London Trauma Conference in June 2011.

## The Pre-hospital Care Programme at Barts and the London School of Medicine and Dentistry, UK

The Pre-hospital Care Programme (PCP) at Barts and the London School of Medicine (BL) was founded as a result of the ambition of one medical student with an interest in pre-hospital medicine. In partnership with key individuals from the London Ambulance Service (LAS), London’s Air Ambulance (LAA) and BL, the student’s experience was developed into a formal academic programme. The structure of the programme is an interlinked, 4 year, student-selected component (SSC), which is entered in the second year of the undergraduate course following an application process and interview. The philosophy behind the programme is one of students gaining experience and training in the field of pre-hospital medicine by way of a spiral curriculum and encouraging teamwork; for each year students are paired up with a mentor and attend shifts with them, responding to emergency calls in London (Table 1). The benefits of one-to-one pairings include allowing learning needs to be tailored to individuals, giving an overview of skills development, fostering teamwork, and the exchange of knowledge between mentor and mentee. As students progress through their medical education, they are increasingly able to contribute with knowledge gained from lectures or hospital experiences. Alongside a minimum number of shifts worked per academic year, students work in partnership with their mentor to complete set learning objectives and, as they grow in competence, may participate in clinical care under the guidance of their mentor. The PCP also runs a monthly Academic Forum (AF) which is open to all interested parties. AFs cover a broad range of topics within pre-hospital care, including clinical cases and presentations from students, paramedics and doctors, as well as LAA led practical sessions.

**Table 1:** Outline of structure of the PCP for undergraduate MBBS students

PCP Year 1 (MBBS Year 2)	6 ambulance shifts with ambulance crew paramedic mentor Plus academic study
PCP Year 2 (MBBS Year 3)	8 shifts on fast response unit with paramedic mentor Plus academic study

PCP Year 3 (MBBS Year 4)	8 physician pre-hospital care shifts with doctor mentor Plus academic study

PCP Year 4 (MBBS Year 5)	Further clinical shifts as appropriate Written project

The pilot for the PCP was launched in November 2007 and since then has grown very quickly. Currently, the PCP can accommodate up to 10 students per year on the undergraduate course, and a specific course for students on the graduate entry course has been developed. AFs are well attended by both medical students and paramedics; the average number of attendees between September 2010 and January 2011 being 120 participants with highly positive feedback received. Due to high levels of interest generated, the PCP has helped create additional, shorter SSCs at BL in pre-hospital care for students who have an interest in the field but are unable to be part of the main PCP.

## National and international perspectives

This PCP has generated interest both nationally and internationally and is currently assisting a number of medical schools in the UK to set up similar programmes. To our knowledge, similar programmes currently exist in some form (or are under construction) at 3 other UK medical schools.

Literature searches using Medline and Embase were performed to identify papers which discussed medical students learning in the pre-hospital environment. Articles were included manually by reviewing abstracts for relevance.

The literature search revealed 10 articles that met the inclusion criteria. There appears to be a paucity of published material regarding pre-hospital medicine and undergraduate medical students. It is likely that more regional programmes may exist, both nationally and internationally, but have not published in the literature. With regards to mandatory pre-hospital education for undergraduates, only one example is described in the UK where a 3 day undergraduate course in pre-hospital medicine was incorporated into the standard curriculum at Birmingham Medical School. Selected students may then continue to pursue their interest in the subject by joining a structured training programme which continued post-graduation [1,2]. Internationally, pre-hospital medicine was incorporated into the fourth year emergency medicine clerkship at the University of Medicine and Dentistry of New Jersey, USA which has been credited with significant improvements in medical student interest and understanding of pre-hospital care. Of note is that the authors found this was achieved “without extraordinary effort” [3]. In Germany 11% of medical schools include an obligatory internship at an ambulance service [4] and data from Frankfurt Medical School indicate that a mandatory pre-hospital course supported by paramedics can serve as valuable training for medical students [5].

In addition, individual students with an interest in pre-hospital care may also participate in pre-hospital based electives with a variety of hospitals and medical services worldwide. Certainly, different locations will have variations in elective structure depending on their area and resources. A 4 month elective emergency medical services (EMS) course for first and second year medical undergraduates was found to have significant effects on both perceived competency and interest in emergency medicine as a career [6].

## The London Trauma Conference Student Day 2011

As a result of increasing interest, PCP students were invited to assist in the organisation of the first student day at the London Trauma Conference (LTC). Aimed at both student doctors and paramedics, it was held on the 22^nd^ June 2011, with a theme of major incident management. The timetable for the day included morning lectures from experts in the field followed by team-based practical skills stations in the afternoon as well as two challenge stations consisting of a triage and a trauma moulage (Table 2). The day was well-received by participants; students were also invited to attend the remainder of the LTC at no extra cost, which was met with a very positive response.

**Table 2:** Outline of LTC student day

Morning lecture topics	Major incidents, a guide for students First on scene for the London bombings Triage and communication Emergency response to earthquakes Urban search and rescue
Afternoon practical sessions	Skills stations: Basic airway management Splints Packaging Haemorrhage control Demonstration of equipment by Hazardous Area Response Team (HART) Team challenges: Triage Trauma

## Conclusion

Since inception the PCP has proven popular with all involved. The authors’ own experiences as students on the programme are that it has greatly improved our confidence to practice alongside practitioners in the pre-hospital environment. We have been very fortunate to learn from experts in different disciplines with a passion for teaching and to observe and participate in clinical care in this unique setting. In addition, the stepwise learning programme delivered by the ambulance service and LAA has enabled us to much more fully appreciate the complexities of emergency patient care. We believe that this experience will make us better doctors when we qualify and will help us greatly in the years after qualification. The fundamentals of pre-hospital care overlap with many key areas of emergency care which are essential for all medical students and with the emergence of pre-hospital emergency medicine as a new subspecialty, we are convinced that there will be both demand and enthusiasm for similar training programmes in other medical schools.

## Competing interests

The authors declare that they have no competing interests.

## Authors’ information

George Kay is a 4^th^ year medical student at Barts and The London School of Medicine and Dentistry and is currently the Lead Medical Student for the PCP.

Enhui Yong and Sara Dahlén are 4^th^ year medical student at Barts and The London School of Medicine and Dentistry.
